# **Reiterating the Importance of Publicly Funded and Provided Primary Healthcare for Non-communicable Diseases: The Case of India** Comment on "Universal Health Coverage for Non-communicable Diseases and Health Equity: Lessons From Australian Primary Healthcare"

**DOI:** 10.34172/ijhpm.2021.137

**Published:** 2021-09-22

**Authors:** Sulakshana Nandi

**Affiliations:** ^1^Public Health Resource Network, Chhattisgarh, India.; ^2^People’s Health Movement, New Delhi, India.

**Keywords:** India, Primary Healthcare, Non-communicable Disease, Health Systems, Social Determinants of Health, Health Equity

## Abstract

India has established health and wellness centres (HWCs) and appointed mid-level healthcare providers (community health officers, CHOs) to provide free and comprehensive primary healthcare (PHC), through screening, prevention, control, management and treatment for non-communicable diseases (NCDs), in addition to existing services for communicable diseases, and reproductive and child health. The range of services being provided and the number of people accessing ambulatory care in these government centres have increased, leading to more equitable healthcare access and financial protection. In policy debates, contestations exist prioritising between PHC or hospital services, and between publicly-provided healthcare or privatised and "purchased" services. Nationally and globally the influence of industries and corporations in health governance has weakened the response against NCDs. PHC initiatives for NCDs must be publicly funded and provided, located within communities, and necessitate action on the determinants of health. The experiences from Australia (a high-income country) and India (a low-and middle-income country) amply illustrate this.

## Introduction

 The article by Fisher et al^1^ on lessons from Australia for universal health coverage (UHC) for non-communicable diseases (NCDs) and health equity, paves way for a much needed discussion on the strategies and policies for comprehensive primary healthcare (PHC) in the context of NCDs, and the continued relevance of the Alma Ata declaration.^2^ Fisher et al^1^ assess the dominant ideas, key actors and interests shaping policy and implementation and financing structures around PHC and NCDs and provide recommendations on PHC design. They highlight how the current model influenced by a biomedical approach, the health industry and medical professionals, is geared towards curative services and episodic care rather than comprehensive PHC, and has services inequitably distributed.^1^ They find that the mix of public and private insurance has adverse implications for health equity, access and finally on health status. Using India’s recent experiences of expanding PHC for NCDs as an illustration, the present commentary argues that the findings and recommendations presented by Fisher et al^1^ are not only relevant to other high-income countries, but also significant for low-income and lower-middle-income countries.^3^

 NCDs and injuries constitute more than one-third of the total disease burden among the poorest one billion people, and a major cause of catastrophic health expenditure for them.^3^ The risks faced by the poor result from their living and work circumstances, such as “inadequate housing and sanitation, polluted environments, infection, food insecurity, unsafe transportation, working conditions, and vulnerable social position,” rather than individual behaviour(p. 13).^3^ NCDs in low-income and lower-middle-income countries are increasing because of exacerbation of these circumstances and getting more recognised due to improved systems for NCD screening and detection.^4^ The burden of communicable diseases and maternal and child health related conditions continue to be high in most countries.

 India too faces a high burden of disease consisting of NCDs, road and other injuries, cancer, communicable diseases, maternal and neonatal conditions and nutritional disorders, disproportionately affecting the poor, rural and historically marginalised communities such as indigenous communities.^5,6^ The rates of undetected and untreated NCDs (hypertension, diabetes), including neglected diseases such as epilepsy, mental illness, sickle-cell disease are extremely high.^5^ High out-of-pocket expenditure, especially on medicines and ambulatory care, pose a burden on households, with the poor facing further impoverishment due to healthcare costs.^7,8^

## Health and Wellness Centres in India: Structures for Implementation

 India initiated reforms in its PHC programme as part of the mandate provided by the National Health Policy (2017) to strengthen PHC systems and invest at least two-thirds of government health spending on it.^6^ After initial pilots, health and wellness centres (HWCs) were inaugurated in February 2018, with the objective to deliver “universal, free comprehensive PHC” through transforming existing health centres (sub-health centres [SHCs] and primary health centres) (p. 18).^6^ By the end of 2022, 150 000 such centres are to be functional. Through the HWC model the range of services being offered by the peripheral health centres was to be expanded to provide screening, prevention, control, management and treatment of chronic diseases, and mental health, palliative and emergency care services, in addition to the previously provided services for communicable diseases and reproductive and child health, all free of cost.^6^ While the emphasis is on ambulatory care, along with a system for referral and continuum of care, there is also a thrust to move away from episodic to more comprehensive healthcare.^6,7^ A new cadre of mid-level healthcare providers known as Community Health Officers (CHOs)^[1]^ consisting primarily of Nurses, have been introduced to provide these services. Various other components of the National Health Mission, such as community health workers (CHWs) (called Accredited Social Health Activists [ASHAs] or Mitanins) and community and women’s collectives, free medicines and diagnostics, free referral transport, and a strengthened secondary care services are to complement the HWCs.^6^

 The experience of the implementation of HWCs over the last three years has lessons for primary level healthcare initiatives aimed at NCDs. As of August 2021^[2]^ more than 76 000 HWCs are reported to be functional. This includes around 4000 urban primary health centres, 21 000 rural primary health centres and more than 50 000 SHCs^[3]^. Additional human resources in the form of around 50 000 CHOs (one for each SHC-level HWC) have been recruited. One SHC normally covers a population of 5000 (3000 in areas having tribal/indigenous populations), therefore the introduction of the additional human resources close to community is very significant.

 States such as Tamil Nadu and Chhattisgarh have seen very similar experiences and outcomes of HWCs, despite having very different demographic and socio-economic profiles. They illustrate the kind of impact such a model can make for equitable access to healthcare and financial protection and in responding to NCDs.

 Tamil Nadu launched the UHC pilot to strengthen PHC services in early 2017, through expanding ambulatory services, introducing screening and management of NCDS, distribution of free drugs, and posting of an additional village health nurse.^9^ SHCs were selected for the intervention as they are closest to community. An evaluation eight months into its implementation found that the share of the private sector in ambulatory care had dropped sharply from the pre-pilot levels, and there was a shift from higher level government facilities to the HWCs, resulting in a huge rise in number of people coming to the government HWCs for services.^9^ This led to a significant reduction in out-of-pocket expenditure, including for transportation and NCD management. Although more women accessed the services, there was an increase in the number of men and people from surrounding villages coming to the centre. Another outcome was that the village health nurses gained confidence in dealing with a range of conditions and improved engagement with the local community.^9^

 More than one third of Chhattisgarh’s population are indigenous people. Historical marginalisation, poverty and inequities in healthcare access have led to higher malnutrition levels, and high mortality and morbidity from communicable diseases such as tuberculosis, malaria, lack of adequate maternal care and increasingly from chronic diseases.^5^ Chhattisgarh introduced HWCs in 2017 and currently nearly 3000 health centres have been converted into HWCs. NCDs were been put high on the HWC mandate and HWCs are responsible for population screening and management of NCDs including follow-up for treatment adherence. Studies find that there has been a big jump in number of people accessing ambulatory care in these government centres, and an increase in the range of services being provided.^10^ While previously primarily reproductive and child health services were provided, now through the HWCs, services for communicable diseases, NCDs and minor emergencies are available. Trained workforce, universal population (above 30 years) screening for NCDs, point-of-care testing, and availability of drugs for NCDs have been introduced to enable this. Follow-up is done within the community by the HWC team that includes the CHWs.^10^ These services are free and available much closer to communities than before, leading to previously excluded rural and “remote” populations including the elderly utilising the services now. Many persons, including men, with NCDs have shifted from accessing care in the private sector to the public sector, resulting in drastic reduction of out-of-pocket expenditure.^10^ However, there is need for improvement in regularity in medicines supplies, and systems for continuum of care and community engagement in all states.^6,7,9,10^

## Contestation Between Private and Public Actors and Interests

 In policy debates nationally, the HWC initiative is caught in a tug-of-war between private and public interests in PHC. There are strong advocates, including within the health ministry, the World Health Organization (WHO), state governments and civil society, who believe that comprehensive PHC initiatives must be publicly funded and publicly provided, for which the government health system has to be strengthened with increased finances and human resources, better infrastructure and systems for procurement and supplies, and higher community involvement.^6,7^ However there is a strong and an opposite push towards prioritising hospital-based services and promoting privatisation and commercialisation of healthcare even within PHC. The main proponents of this are the healthcare industry, and the national government, supported by the NITI Aayog, National Health Authority and other pro-market agencies such as the World Bank, philanthrocapitalists and private consultancy agencies. They advocate “strategic purchasing” (mainly from the private sector) for PHC in which the government is expected to perform the role of stewardship rather than provide services on its own.^11^ This model exists in the second component of Ayushman Bharat, the Pradhan Mantri Jan Arogya Yojana (PMJAY) which is for hospitalised care. “Purchasing” healthcare from the private sector has led to inequitable utilisation of healthcare, diversion of public funds to the private sector (more than 75% of the PMJAY funds go to the private sector), weakening of public hospitals and commercialisation of healthcare.^12^ Replicating this in PHC will be disastrous for people and communities, especially for those who are most vulnerable.^8^

## Action on Social Determinants

 Even in India it is amply clear that the response to the NCD crisis must include policies and interventions within the healthcare system and on food/nutrition, tobacco, pollution and other determinants.^13^ In Chhattisgarh CHWs (called ASHAs or Mitanins) have successfully undertaken action on SDH along with health committees at the community level.^14^ Such action would not have been possible within a privatised health system. Nationally, though some work has been initiated on improving mental health services and legislating tobacco control, in recent years India’s policy environment has provided an impetus to industries and corporations, and weakened labour regulations and environment protection laws. Changes recently made to agricultural legislation could have dire consequences for environment, agricultural sustainability, food systems and nutrition.^15^ The impact of government’s pro-industry policies on health and its determinants are visible on the most vulnerable group in society, the children. Government’s own surveys show that malnutrition levels among children have increased in many states.^16^

## Lessons Learnt and The Way Forward

 While the economic, socio-cultural, epidemiological, health systems and policy-making contexts differ greatly between Australia and India, some common lessons emerge. PHC systems that are organized involving health workers (who may not be physicians), having community-based healthcare services with integrated follow-up and referral systems, regular supply of medicines, and incorporating inter-sector collaboration, and community participation are better and more equitable.^1,4,6,7,9,10^ There have been encouraging efforts towards comprehensive PHC programmes through public health systems.^4,6,7,9,10^ Privatised and insurance-based systems of PHC make services urban-centric and focused on episodic care.^1,4^ They also fail to address necessary continuum of care and preventive and promotive dimensions of NCDs.^1,4^ However the influence of industries and corporations in health governance has weakened the response against NCDs both globally and nationally. Within the mainstream UHC discourse PHC is relegated to the position of primary level healthcare and the role of the government has been perverted towards “strategic purchasing” from the private sector.^2^ This has adverse implications for PHC and health equity. Governments and the global health community must act urgently to counter the influence of industries and corporations and uphold the right to health of all people. Solidarities must be forged between academics, civil society and social movements to highlight the commercial determinants of health and advocate for comprehensive PHC. PHC initiatives for NCDs must be publicly funded and provided, located within communities to address their needs (both hidden and explicit), necessitate action on the socio-economic, political, environmental and commercial determinants of health and be designed to ensure that the poorest who are most at risk of disease and death should not be excluded. The experiences from Australia (a high-income country) and India (a low- and middle-income country) amply illustrate this.

## Ethical issues

 Not applicable.

## Competing interests

 Author declares that she has no competing interests.

## Author’s contribution

 SN is the single author of the paper.

## Endnotes

 [1] CHOs are primarily nursing graduates who have undergone a six-month bridge course on their role as mid-level care providers in SHC-level HWCs. [2] Data on the number of HWCs (national and state-wise) is available at the website of Ayushman Bharat – Health and Wellness Centres; https://ab-hwc.nhp.gov.in. [3] Primary Health Centers are located at 30 000 population (20 000 for tribal/indigenous areas) and are supposed to have a physician and in-patient facility (around 6 beds). There are urban and rural primary health centers; SHCs is the lowest physical health unit, located at 5000 population (3000 in areas having tribal/indigenous populations).
